# Efficacy and Safety of Fuzheng Yiqi Kang-Ai Decoction Combined with External Irradiation in the Treatment of Undifferentiated Thyroid Carcinoma and Its Influence on Antiangiogenesis

**DOI:** 10.1155/2022/3589924

**Published:** 2022-05-16

**Authors:** Li Bian, Jing Zhang, Pengbo Wang

**Affiliations:** ^1^Ministry of Foreign Affairs of Science and Education, Shandong Cancer Hospital and Institute, Shandong First Medical University and Shandong Academy of Medical Sciences, Jinan, 250117 Shandong Province, China; ^2^Oncology Department I, Zibo Central Hospital, Zibo, 255020 Shandong Province, China; ^3^Radiotherapy Department I, Yantaishan Hospital, Yantai City, 264000 Shandong Province, China

## Abstract

**Objective:**

To explore the efficacy and safety of Fuzheng Yiqi Kang-ai (FZYQKA for short) decoction with external irradiation in the treatment of undifferentiated thyroid carcinoma (UTC) and its influence on antiangiogenesis*. Methods.* In this retrospective study, the clinical data of 120 patients with UTC admitted to Zibo Central Hospital (February 2019-February 2020) were retrospectively analyzed, and the patients were equally divided into the experimental group (EG) and the control group (CG) according to the order of admission. All patients received external irradiation, and the EG received FZYQKA decoction additionally. FZYQKA decoction was taken orally 1 dose daily in 3 times with a total of 100 ml, for a total of 2 months. Short-term efficacy, incidence of acute radiotoxic responses, levels of matrix metalloproteinases (MMPs), indexes of immune function, and level of vascular endothelial growth factor (VEGF) were compared between both groups.

**Results:**

Compared with the CG, the disease control rate of the EG was obviously higher (73.3% vs. 40.0%, *P* < 0.001). The acute radiotoxic responses of the two groups were mainly grade I-II oral mucositis, radiodermatitis, pharyngitis, esophagitis, and myelosuppression, and only three patients (5.0%) had grade III-IV toxic reactions. Compared with the CG, the incidence of grade I-II oral mucositis, radiodermatitis, pharyngitis, and esophagitis in the EG was obviously lower (*P* < 0.05). After treatment, compared with the CG, levels of MMPs and VEGF of the EG were obviously lower (*P* < 0.001). After treatment, compared with the CG, indexes of immune function of the EG were obviously higher (*P* < 0.001).

**Conclusion:**

For patients with UTC, FZYQKA decoction combined with external irradiation can exert the antiangiogenesis effect, reduce levels of MMPs, and optimize the short-term efficacy. The safe treatment method has mild toxic and side effects, which should be popularized in practice.

## 1. Introduction

Thyroid carcinoma is the most common malignant solid tumor of endocrine system, of which undifferentiated thyroid carcinoma (UTC) accounts for 5%-10% [1, 2], and UTC has incomparable severity and invasive ability in thyroid carcinoma [3]. Patients usually present with hoarseness, cough, and dysphagia and are prone to have local fatal invasion and distant metastasis [4–7]. In recent years, the concept of holism and the dialectical theory of Traditional Chinese medicine (TCM) has achieved remarkable results in the treatment of various cancers [8–10]. Scholars Sun and Respiration applied Fuzheng Yiqi Kang-ai (FZYQKA for short) decoction in patients with advanced non-small-cell carcinoma, which found that the levels of vascular endothelial growth factors (VEGFs) were remarkably reduced, while levels of matrix metalloproteinases (MMPs) were greatly increased [11]. The high expression of MMPs is associated with invasion, metastasis, and poor prognosis of tumors, indicating that the drug can reduce the possibility of distant metastasis of lung cancer and reduce the invasive ability of malignant tumors. The FZYQKA decoction contains Mongolian milkvetch root, largehead atractylodes rhizome, tuckahoe, cassia twig, and other herbs. Among them, tuckahoe and cassia twig can downregulate HIF-1*α* expression in lesions and then inhibit the combination of HIF-1*α* and VEGF under hypoxia, decrease the rate of angiogenesis by regulating the HIF-1*α*-VEGF signaling pathway, and avoid the acceleration of tumor growth through transcription and posttranscriptional control after HIF-1 binds to target genes. Pinellia tuber, Fritillaria, and Chinese peony also have the effect of antiangiogenesis [12], which is speculated to be the important mechanism of reducing the VEGF level in patients with advanced non-small-cell carcinoma. According to the previous literature, no scholars have applied FZYQKA decoction in the treatment of UTC, but the positive role of this drug in the treatment of other cancers has been confirmed. Therefore, this paper combined FZYQKA decoction with external irradiation to explore the efficacy and safety of the combined method in the treatment of UTC and its influence on antiangiogenesis.

## 2. Materials and Methods

### 2.1. Research Design

This retrospective study was performed in the Zibo Central Hospital (February 2019-February 2020) to explore the efficacy and safety of FZYQKA decoction combined with external irradiation in the treatment of UTC and its influence on antiangiogenesis.

### 2.2. Research Subjects

Clinical data of 120 patients with UTC admitted to Zibo Central Hospital (February 2019-February 2020) were retrospectively analyzed. Inclusion criteria were as follows. (1) After pathological and imaging examination, patients were diagnosed with UTC [13]. (2) Patients received the whole treatment in the hospital, and no one died, transferred halfway, and stopped treatment. (3) The clinical data of patients were complete. Exclusion criteria were as follows. (1) Patients had psychiatric diseases or could not be communicated with. (2) Patients withdrew the experiment halfway. (3) Patients had severe heart, brain, liver, and kidney dysfunction and were complicated with other malignant tumors.

### 2.3. Procedures

A total of 120 patients were included in this study and equally divided into the experimental group (EG) and the control group (CG) according to the order of admission. On the day that the patients agreed to participate in the study, the study group collected sociodemographic data and clinical data. After analysis, no obvious difference was found in general data between both groups (*P* > 0.05) (see Table 1).

### 2.4. Moral Consideration

The study conformed to the principles of Declaration of Helsinki [14] and was approved by the Ethics Committee of the Zibo Central Hospital. After enrollment, patients were informed of the purpose, significance, content, and confidentiality of the experiment by the study group.

### 2.5. Withdrawal Criteria

For patients who were in the following situations and inappropriate to continue the experiment according to the research group, their medical record sheets were kept, but the data was not analyzed. (1) Patients experienced adverse events or serious adverse events. (2) Patients presented with deterioration during the experiment. (3) Patients had severe comorbidities or complications. (4) Patients were not willing to continue the clinical trial and asked the study group for withdrawal.

### 2.6. Methods

External irradiation was performed with 6 MC X-ray, three dimensional conformal radiotherapy (3-DCRT), or intensity modulated radiation therapy (IMRT). Varian Trilogy accelerator [NMPA (I) 20152062178] was used for treatment. The dose fractionation was as follows: (1) patients with recurrent UTC received IMRT with 50-60 Gy/25-28 times (1.8-2 Gy/time) and (2) patients with metastatic UTC. Patients with bone metastasis received 3-DCRT or IMRT with 24-50 Gy/25-28 times (1.8-4 Gy/time). Patients with liver, brain, and lung metastasis received stereotactic radiotherapy (SRT) with 48-64 Gy/7-8 times (6-8 Gy/time).

The EG was treated with the FZYQKA decoction additionally, and the prescription was as follows: Astragalus membranaceus 30 g, largehead atractylodes 30 g, Poria cocos 30 g, Codonopsis pilosula 25 g, Rehmannia glutinosa 25 g, lily 25 g, dried tangerine peel 15 g, Pinellia ternata 15 g, Fritillaria 10 g, almond 10 g, guar 10 g, Angelica sinensis 10 g, Chinese peony 10 g, Radix Bupleuri 10 g, Cinnamomi ramulus 5 g, Fructus Aurantii Immaturus 5 g, Schisandra 5 g, licorice 5 g, jujube dates 5 g, and fresh ginger 5 g. FZYQKA decoction was taken orally 1 dose daily in 3 times with a total of 100 ml, for a total of 2 months.

### 2.7. Observation Criteria

1. General data. The general data table was fulfilled by patients themselves
2. Short-term efficacy. According to the Response Evaluation Criteria in Solid Tumors (RECIST) [15], the patients' condition was divided into complete response (CR), partial response (PR), stable disease (SD), and progressive disease (PD). CR + PR = Objective Response Rate (ORR). CR + PR + SD = Disease Control Rate (DCR). The therapeutic effect of the patients was compared
3. Incidence of acute radiotoxic responses. Acute radiotoxic responses of patients were recorded in line with the Performance and Evaluation Criteria for Acute and Subacute Toxicity [16] by the World Health Organization (WHO)
4. Levels of MMPs. Serum MMP-2 and MMP-9 levels were measured before treatment (*T*_1_), 1 month after treatment (*T*_2_), and 2 months after treatment (*T*_3_)
5. Indexes of immune function. Levels of NK, CD4^+^ Th17, CD4^+^CD^+^25Treg, and Th17/Treg were detected by a flow cytometer (ACEA Biosciences Inc.; Zhejiang Medical Products Administration Certified No. 20142400581) at *T*_1_, *T*_2_, and *T*_3_
6. VEGF level. The VEGF level was detected at *T*_1_, *T*_2_, and *T*_3_

### 2.8. Statistical Processing

In this study, the data were processed by SPSS20.0 and graphed by GraphPad Prism 7 (GraphPad Software, San Diego, USA). Including enumeration data and measurement data, the study used *X*^2^ test and *t* test. The differences were statistically significant at *P* < 0.05.

## 3. Results

### 3.1. Comparison of General Data of Patients

No significant difference was found in the general data of patients between both groups (*P* > 0.05) (see Table 1).

### 3.2. Comparison of Short-Term Efficacy

Compared with the CG, DCR in the EG was obviously higher (*P* < 0.001) (see Table 2).

### 3.3. Comparison of Incidence of Acute Radiotoxic Responses

The acute radiotoxic responses of the two groups were mainly grade I-II oral mucositis, radiodermatitis, pharyngitis, esophagitis, and myelosuppression, and only 3 patients had grade III-IV toxic reactions. Compared with the CG, the incidence of grade I-II oral mucositis, radiodermatitis, pharyngitis, and esophagitis in the EG was obviously lower (*P* < 0.05) (see Table 3).

### 3.4. Comparison of Levels of MMPs

**Figure 1:**
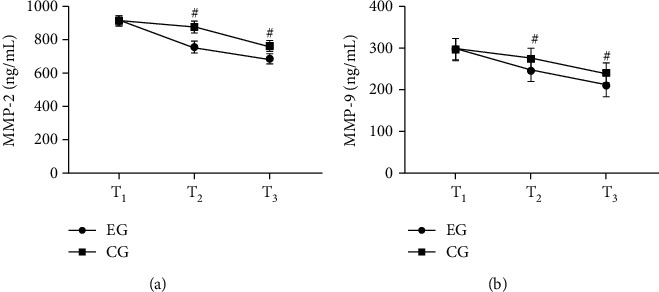
Comparison of levels of MMPs ($\overset{¯}{x}\pm s$, ng/mL). Note: in (a) and (b), the abscissa was *T*_1_, *T*_2_, and *T*_3_ from left to right, respectively. The line with dots was the EG, and the line with squares was the CG. # indicated *P* < 0.001.

After treatment, compared with the CG, levels of MMPs of the EG were obviously lower (*P* < 0.001) (see Figure 1).

Figure 1(a) was the MMP-2. No obvious difference was observed in MMP-2 at *T*_1_ in both groups (912.65 ± 30.15 vs. 913.68 ± 30.65, *P* > 0.05). Compared with the CG, MMP-2 at *T*_2_ and *T*_3_ in the EG was obviously lower (756.21 ± 35.68 vs. 876.68 ± 35.21 and 685.98 ± 30.50 vs. 762.68 ± 32.68, *P* < 0.001).

Figure 1(b) was the MMP-9. No obvious difference was observed in MMP-9 at *T*_1_ in both groups (297.65 ± 25.65 vs. 296.68 ± 26.85, *P* > 0.05). Compared with the CG, MMP-9 at *T*_2_ and *T*_3_ in the EG was obviously higher (245.98 ± 25.68 vs. 274.35 ± 25.41 and 210.58 ± 26.98 vs. 240.98 ± 23.98, *P* < 0.001).

### 3.5. Comparison of Indexes of Immune Function

**Figure 2:**
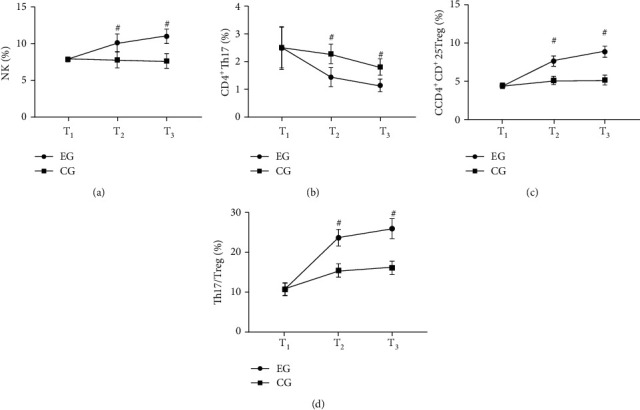
Comparison of indexes of immune function ($\overset{¯}{x}\pm s$, %). Note: in (a)–(d), the abscissa was *T*_1_, *T*_2_, and *T*_3_ from left to right, respectively. The line with dots was the EG, and the line with squares was the CG. # indicated *P* < 0.001.

After treatment, compared with the CG, indexes of immune function of the EG were obviously higher (*P* < 0.001) (see Figure 2).

Figure 2(a) was NK. No obvious difference was observed in NK at *T*_1_ in both groups (7.86 ± 0.31 vs. 7.87 ± 0.30, *P* > 0.05). Compared with the CG, NK at *T*_2_ and *T*_3_ in the EG was obviously higher (10.14 ± 1.20 vs. 7.80 ± 1.08 and 11.02 ± 0.98 vs. 7.65 ± 1.00, *P* < 0.001).

Figure 2(b) was CD4^+^ Th17. No obvious difference was observed in CD4^+^ Th17 at *T*_1_ in both groups (2.51 ± 0.78 vs. 2.53 ± 0.74, *P* > 0.05). Compared with the CG, CD4^+^ Th17 at *T*_2_ and *T*_3_ in the EG was obviously higher (1.45 ± 0.35 vs. 2.30 ± 0.36 and 1.15 ± 0.23 vs. 1.82 ± 0.30, *P* < 0.001).

Figure 2(c) was CD4^+^CD^+^25Treg. No obvious difference was observed in CD4^+^CD^+^25Treg at *T*_1_ in both groups (4.41 ± 0.35 vs. 4.43 ± 0.36, *P* > 0.05). Compared with the CG, CD4^+^CD^+^25Treg at *T*_2_ and *T*_3_ in the EG was obviously higher (7.65 ± 0.68 vs. 5.11 ± 0.54 and 8.89 ± 0.74 vs. 5.16 ± 0.65, *P* < 0.05).

Figure 2(d) was Th17/Treg. No obvious difference was observed in Th17/Treg at *T*_1_ in both groups (10.68 ± 1.65 vs. 10.70 ± 1.54, *P* > 0.05). Compared with the CG, Th17/Treg at *T*_2_ and *T*_3_ in the EG was obviously higher (23.68 ± 2.10 vs. 15.44 ± 1.69 and 25.98 ± 2.54 vs. 16.10 ± 1.68, *P* < 0.05).

### 3.6. Comparison of Patients' VEGF Levels

At *T*_1_, no statistical difference in VAGF (pg/mL) levels between EG and CG was observed (27.65 ± 2.14 vs. 27.44 ± 2.10, *P* > 0.05); and at *T*_2_ and *T*_3_, the VEGF levels were obviously lower in EG than in CG (15.98 ± 1.65 vs. 20.65 ± 1.77 and 14.10 ± 1.65 vs. 18.68 ± 1.78, *P* < 0.001).

## 4. Discussion

Thyroid carcinoma arises from the thyroid gland [17], and UTC is of the highest degree of malignancy [18]. Patients present with invasion and metastasis at an early stage, and the diameter of the tumor is observed as above 5 cm at the time of diagnosis [19]. Due to generally poor prognosis, rapid treatment measures should be taken once diagnosed so as to improve the survival period of patients. Because UTC is closely related to thyroid stimulating hormone, there is a high prevalence of p53 mutations [20]. With low sensitivity of conventional chemotherapeutic drugs, only less than 18% of patients have a weak reaction to clinical common drugs such as doxorubicin [21]. Nowadays, with the continuous upgrading of radiotherapy technology, external irradiation has been used in the treatment of UTC. Mild toxic reaction is shown in some studies, which is conducive to relieving the clinical symptoms of patients. In this study, it was found that the acute radiotoxic responses of the two groups were mainly grade I-II oral mucositis, radiodermatitis, pharyngitis, esophagitis, and myelosuppression, and only 3 patients had grade III-IV toxic reactions, which indicated that external irradiation had ideal safety and could ensure the quality of life of patients. However, radiotherapy is the palliative care, which is usually effective only in a short term. Sensitivity still fails to make 80% of patients obtain long-term therapeutic effect [22]. A large number of international literature have confirmed that radiotherapy alone has no remarkable effect on the long-term survival rate of patients with UTC [23], so it is essential to adopt other treatment methods.

Recently, the advantages of TCM in the treatment of malignant tumors have become increasingly prominent. It has been confirmed that self-made Shugan Sanjie Qudu decoction, Jiawei Suanzaoren decoction, and self-made Chaizhi decoction can relieve the clinical symptoms of thyroid carcinoma [24], improve organismal tolerance, and enhance the comprehensive treatment effect. TCM believes that patients with thyroid carcinoma have Yin and Yang disorders due to disorder of Qi and dysfunction of Zang-fu organs. Meanwhile, the disease is characterized by deficiency in the Ben (root) and excess in the Biao (branch), which results in Qi-yin deficiency and especially toxin retention and Qi and Yin deficiency in patients with UTC, so FZYQKA decoction should be used in the treatment. Astragalus membranaceus, largehead atractylodes, Poria cocos, and Codonopsis pilosula in FZYQKA decoction have the effect of invigorating Qi and can inhibit the metastasis and invasion of malignant tumors. Therefore, compared with the CG, levels of MMPs in the EG after treatment were obviously lower (*P* < 0.001), indicating that the invasion ability of UTC was obviously reduced. In addition, Astragalus polysaccharide can also improve the immune function of patients and especially the regulation of T-lymphocyte subsets, and Poria cocos can also improve the nonspecific immunity. The combination of all herbal medicine can effectively improve the immunity of patients. Therefore, compared with the CG, indexes of immune function of the EG after treatment were conspicuously higher (*P* < 0.001).

Meanwhile, the growth of thyroid cancer is closely related to VEGF, and the positive expression rate of VEGF in thyroid cancer with different pathological types and clinical stages is significantly different, indicating that VEGF is an important marker of active biological behavior of thyroid cancer cells. VEGF can elevate tumor vascular permeability and create a fibrin matrix structural basis for stromal invasion, while multiple herbs in FZYQKA decoction have antiangiogenic effects. In the formula, Pinellia tuber contains rich baicalein, a substance that is able to downregulate VEGF level and upregulate Rb-1 expression, resulting in inhibition of tumor angiogenesis, and the stigmasterol in Pinellia tuber can destroy the TNF-*α*-VEGFR-2 axis and then inhibit the proliferation of endothelial cells. Paniculate bolbostemma can also induce the apoptosis of endothelial cells, inhibit VEGF expression, and reduce capillary vessel density, and cassia twig and tuckahoe can inhibit the VEGF expression under hypoxia. On this basis, Chinese Angelica and tuckahoe can further exert the effect of nourishing blood. Chinese thorowax root, cassia twig, immature orange fruit, dried tangerine peel, and other herbs have the effects of removing blood stasis and resolving hard mass, and in combination with Rehmannia root, lily bulb, Fritillaria, bitter apricot seed, etc., the effects of clearing heat and promoting diuresis as well as removing blood stasis and expelling pus can be exerted. The decoction can remarkably improve the short-term efficacy of patients. The research of scholars Huiyun et al. shows that Saikosaponin D in Radix Bupleuri can inhibit the activity of malignant tumor cells and improve the induction of apoptosis. At the same time, Saikosaponin A can inhibit the growth of cancer cells and the metastasis and invasion of cancer cells, which is conducive to controlling the development of UTC. Therefore, the short-term efficacy of patients with pancreatic cancer treated with Radix Bupleuri has been obviously improved [25]. This study also found that compared with the CG, the DCR of the EG was conspicuously higher (*P* < 0.001), indicating that FZYQKA decoction could work on UTC and improve the comprehensive effect of external irradiation.

In conclusion, for patients with UTC, FZYQKA decoction combined with external irradiation can exert the effect of antiangiogenesis, reduce levels of MMPs, and optimize the short-term efficacy. The safe treatment method has mild toxic and side effects, which should be popularized in practice.

## Figures and Tables

**Table 1:** Comparison of general data.

Group	EG (*n* = 60)	CG (*n* = 60)	*X* ^2^/*t*	*P*
Gender			0.034	0.853
Male	25	26		
Female	35	34		
Age (years old)				
Range	30-76	33-74		
Average age	56.21 ± 5.68	56.28 ± 5.10	0.071	0.944
Mean body mass (kg)	56.98 ± 2.68	56.89 ± 2.54	0.189	0.851
BMI (kg/m^2^)	22.58 ± 1.65	22.62 ± 1.70	0.131	0.896
Pathological stages				
I	20	22	0.147	0.702
II	24	23	0.035	0.852
III	13	10	0.484	0.487
IV	3	5	0.536	0.464
Marital status			0.154	0.695
Married	42	40		
Unmarried, divorced, or widowed	18	20		
Metastasis				
Pulmonary metastasis	40	42	0.154	0.695
Bone metastasis	2	3	0.209	0.648
Brain metastasis	1	1	0.000	1.000
Liver metastasis	2	1	0.342	0.559
Location of tumors			0.436	0.509
Unilateral	56	54		
Bilateral	4	6		
Living habits				
Smoking history	32	30	0.134	0.715
History of drinking	26	28	0.135	0.714
Monthly income (yuan)			0.035	0.853
≥4000	25	24		
<4000	35	36		
Medical payment				
Medical insurance	32	33	0.034	0.855
Commercial insurance	20	20	0.000	1.000
Public medical care	8	7	0.076	0.783
Education degree			0.035	0.852
High school degree and below	36	37		
University degree and above	24	23		

**Table 2:** Comparison of short-term efficacy [*n* (%)].

Group	Complete response (CR)	Partial response (PR)	Stable disease (SD)	Progressive disease (PD)	Objective response rate (ORR)	Disease control rate (DCR)
EG	2 (3.3)	18 (30.0)	24 (40.0)	16 (26.7)	20 (33.3)	44 (73.3)
CG	0 (0.0)	12 (20.0)	12 (20.0)	36 (60.0)	12 (20.0)	24 (40.0)
*X* ^2^	2.034	1.600	5.714	13.575	2.727	13.575
*P*	0.154	0.206	0.017	0.000	0.099	0.000

**Table 3:** Comparison of incidence of acute radiotoxic responses [*n* (%)].

Group	EG (*n* = 60)	CG (*n* = 60)	*X* ^2^	*P*
Oral mucositis				
I-II	30 (50.0)	42 (70.0)	5.000	0.025
III-IV	0 (0.0)	1 (1.7)	1.008	0.315
Radiodermatitis				
I-II	25 (41.7)	36 (60.0)	4.035	0.045
III-IV	0 (0.0)	2 (3.3)	2.034	0.154
Pharyngitis and esophagitis				
I-II	24 (40.0)	35 (58.3)	4.035	0.045
III-IV	0 (0.0)	0 (0.0)	—	—
Myelosuppression				
I-II	6 (10.0)	10 (16.7)	1.154	0.283
III-IV	0 (0.0)	0 (0.0)	—	—

## Data Availability

Data to support the findings of this study is available on reasonable request from the corresponding author.
